# Molecular Study of a *Hoxa2* Gain-of-Function in Chondrogenesis: A Model of Idiopathic Proportionate Short Stature

**DOI:** 10.3390/ijms141020386

**Published:** 2013-10-14

**Authors:** Pierre M. L. Deprez, Miloud G. Nichane, Benoît G. Lengelé, René Rezsöhazy, Catherine Nyssen-Behets

**Affiliations:** 1 Ecole de Kinésiologie et Récréologie, Faculté des Sciences de la Santé et Services Communautaires, Université de Moncton, Moncton, NB E1A 3E9, Canada; E-Mail: pierre.deprez@umoncton.ca; 2 Embryologie Moléculaire et Cellulaire Animale, Institut des Sciences de la Vie, Université catholique de Louvain, Louvain-la-Neuve 1348, Belgium; E-Mails: miloud.nichane@uclouvain.be (M.G.N.); rene.rezsohazy@uclouvain.be (R.R.); 3 Pôle de Morphologie, Institut de Recherche Expérimentale et Clinique, Université catholique de Louvain, Brussels 1200, Belgium; E-Mail: benoit.lengele@uclouvain.be

**Keywords:** proportionate short stature, endochondral ossification, Hoxa2, chondrogenesis

## Abstract

In a previous study using transgenic mice ectopically expressing *Hoxa2* during chondrogenesis, we associated the animal phenotype to human idiopathic proportionate short stature. Our analysis showed that this overall size reduction was correlated with a negative influence of *Hoxa2* at the first step of endochondral ossification. However, the molecular pathways leading to such phenotype are still unknown. Using protein immunodetection and histological techniques comparing transgenic mice to controls, we show here that the persistent expression of *Hoxa2* in chondrogenic territories provokes a general down-regulation of the main factors controlling the differentiation cascade, such as Bapx1, Bmp7, Bmpr1a, Ihh, Msx1, Pax9, Sox6, Sox9 and Wnt5a. These data confirm the impairment of chondrogenic differentiation by *Hoxa2* overexpression. They also show a selective effect of *Hoxa2* on endochondral ossification processes since Gdf5 and Gdf10, and Bmp4 or PthrP were up-regulated and unmodified, respectively. Since *Hoxa2* deregulation in mice induces a proportionate short stature phenotype mimicking human idiopathic conditions, our results give an insight into understanding proportionate short stature pathogenesis by highlighting molecular factors whose combined deregulation may be involved in such a disease.

## 1. Introduction

Proportionate short stature (PSS) is a growth-related disease that is characterized by a severe reduction in the length of the trunk and limbs, resulting in adult patients with smaller but proportional body parts [1]. The main causes of short stature are well known and can be classified as either primary (associated with Turner syndrome, osteogenesis imperfecta or IGF-I deficit, for example) or secondary (a consequence of malnutrition, growth hormone deficiency or irradiation, for example). However, idiopathic cases of short stature are still often reported [2].

While characterizing a *Hoxa2* gain-of-function transgenic mouse model in order to study the functional relationship between *Hox* genes activity and the control of chondrogenic differentiation, we observed that the persistent and ectopic activity of *Hoxa2* in cartilage led to a reduction of ossification centers. This reduction impacted the endochondral ossification process, resulting in a postnatal growth defect mimicking human PSS [3,4]. Indeed, whereas the transgenic mice were similar in size to the control animals at birth, they featured a significant but harmonious shortening of both the trunk and the appendicular skeleton as early as the day four. We determined that this reduced length phenotype in a mouse skeleton was not due to gross proliferation, apoptosis or endochondral ossification impairments, but was rather related to a decrease in the number of cells entering chondrogenesis [4]. The diagnostic approach for short stature [2] helped associate this mouse phenotype to idiopathic PSS.

Skeletogenesis is a developmental process consisting of two main differentiation pathways in which undifferentiated mesenchymal cells first differentiate into osteoblasts or chondrocytes to later enter osteogenesis or chondrogenesis, respectively. While the former implies direct formation of bone and involves multiple maturation steps leading the osteoblast to become a mature osteocyte [5,6], the latter leads the chondrocyte to proliferate, to hypertrophy and then, after physiological death, leave empty lacunae for osteoblast deposition through vascular invasion [7,8]. These developmental pathways are called intramembranous (or dermal) and endochondral ossification, respectively.

The molecular control of endochondral ossification is quite complex. After condensation, mesenchymal cells differentiate into chondrocytes through the induction of the major regulator of chondrogenesis, *Sox9* [9]. *Sox9* induction relies on Pax1 and Pax9 through Bapx1 stimulation [10]. During its first step, chondrogenesis is also stimulated by Msx1, Msx2, as well as Wnt5a, Bmp7 and Bmp4 signaling [11–14]. Afterwards, a positive regulatory feedback loop between *Sox9* and *Bapx1* induces cellular proliferation [15]. Sox9 maintains chondrocyte proliferation with the help of its partners, Sox5 and Sox6. Paracrine influences support this cell multiplication process by PthrP, Ihh, Bmp7 and Bmpr1a [16,17]. Next, *Sox9* is inhibited by Wnt5a through β-catenin [18]. This allows for maturation into hypertrophic chondrocytes. This maturation step is also stimulated by Bmpr1a activation as well as by Runx2, and Msx2 transcription factors [19–21]. Finally, *Runx2* expression brings hypertrophic chondrocytes to physiological death and triggers osteogenesis [22]. In this regard, it has been suggested that *Runx2* is inhibited by Sox9 through Bapx1 stimulation during hypertrophy [23].

While we previously showed in a gain-of-function transgenic mouse model that Hoxa2 may impact on the mesenchymal-to-chondrocyte differentiation step and give rise to a proportionate short stature phenotype [4], we show here that several molecular regulators of endochondral ossification are significantly decreased by a *Hoxa2* gain-of-function in chondrogenesis.

## 2. Results and Discussion

In 2007, Massip and colleagues generated a transgenic mouse model which induced *Hoxa2* gene expression in a Cre-mediated way [3]. Crossing these mice with *Col2a1-Cre* transgenics [24] provoked an ectopic and persistent expression of *Hoxa2* in every *Col2a1* expressing cell, *i.e.*, in all cells initiating chondrogenesis. The resulting *Col2a1/Hoxa2-lacZ* mice displayed cartilage defects as E18.5 embryos featured a reduction of ossification centers in their skeleton. In-depth analysis of their postnatal phenotype revealed they could be used as a suitable model for the study of the idiopathic PSS in humans considering the proportionate small body size of the animals could not be associated with syndromes, pathologies or other known disorders [4]. By analyzing the impact of *Hoxa2* on chondrogenesis, we excluded any significant impairment on the proliferation rate, apoptosis, maturation rate and ultrastructure of chondrocytes. Instead, it appeared that the expression of *Hoxa2* at the onset of chondrogenesis exerts an inhibitory influence on the mesenchymal-to-chondrocyte differentiation, particularly via a decreased number of cells entering differentiation [4]. These data were consistent with previous observations which showed that *Hoxa2* expression is detected in every mesenchymal cell of the future cartilaginous template of the second branchial arch derivatives. However, its expression is no longer detected as soon as chondrogenesis starts, *i.e.*, concomitant with *Sox9* expression [25].

**Figure 1:**
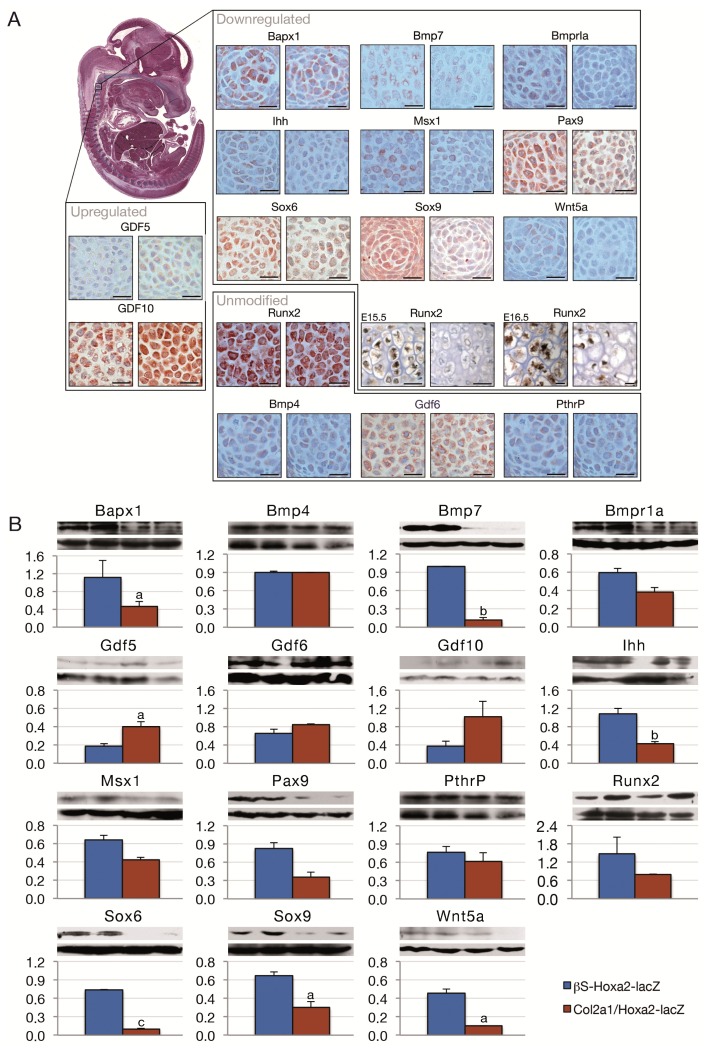
Immunohistochemistry (**A**) and western blotting (**B**) on E13.5 *βS-Hoxa2-lacZ* (left) and *Col2a1/Hoxa2-lacZ* (right) vertebral bodies. (**A**) Runx2 was unmodified at E13.5 but reduced in pre- and hypertrophic chondrocytes as shown in E15.5 and E16.5 limbs, respectively; (**B**) Immunoblots are featured as follows (from top to bottom): the targeted molecule, β-actin as a control and the semi-quantification results using mean gray values. (a) *p* < 0.05; (b) *p* < 0.005 and (c) *p* < 0.0005. Magnification 40×. Scale bar = 0.1 cm. *n* = 5.

In order to understand the molecular disruption that takes place in the Hoxa2-induced short stature phenotype, embryos carrying both transgenes (*Col2a1/Hoxa2-lacZ*) or only the *βS-Hoxa2-lacZ* transgene were processed for immunohistochemistry and western blotting towards several regulators of skeletal development. Indeed, when deregulated, some of regulators induce similar phenotypical characteristics to our transgenic model (see below). For example, while *Bapx1* knockout mice feature an absence of ossification centers in their cervical vertebrae [26], *Bmp7* null animals present a severe postnatal size reduction [27]. We compared the expression level of these factors using tissue immunostaining in the vertebrae of *Col2a1/Hoxa2-lacZ* and *βS-Hoxa2-lacZ* E13.5 embryos because almost every part of the skeleton is under development at this stage (Figure 1A). After total excision, the spine of E13.5 mutants and controls was processed for western blotting and semi-quantification of western blot signals was carried out (Figure 1B).

**Figure 2:**
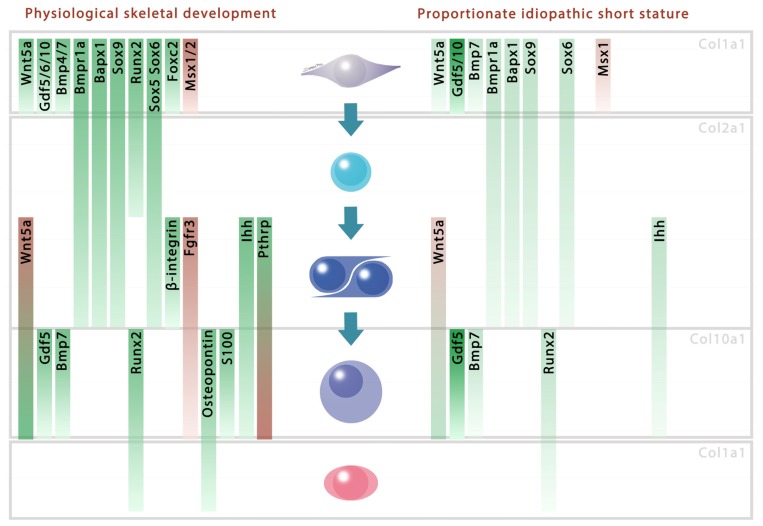
Model featuring the molecular deregulation induced by *Hoxa2* over-expression and associated with a proposed mechanism leading to idiopathic proportionate short stature. Molecular expressions are featured with their function and interactions on the left panel according to the mesenchymal (Col1a1, upper quadrant), differentiating (Col2a1, middle quadrant) and hypertrophic (Col10a1, lower quadrant) stages. Molecules are presented with their known interactions according to the literature [11,15,17,18,28,29,31–40]. Molecules that present a negative or positive influence on differentiation are shown in red and green, respectively (left panel). The factors that were reduced or increased in their protein levels in *Col2a1/Hoxa2-lacZ* are featured with red or green arrows, respectively (right panel). The stars indicate a significant difference observed using western blotting semi-quantification.

We showed that mice with an ectopic expression of *Hoxa2* feature decreased Bapx1, Bmp7, Bmpr1a, Ihh, Msx1, Pax9, Sox6, Sox9 and Wnt5a and increased Gdf5 and Gdf10 protein levels. When significant, the protein level reductions reached up to one eighth of the control ones. The other molecules showed similar patterns for both immunohistochemistry and western blots (Figure 1). This is interesting since most of the decreased proteins stimulate the mesenchymal-to-chondrocyte transition [10,11,15,28–31] (Figure 2, left panel). This is consistent with the observation that continued *Hoxa2* expression impairs the early differentiation of mesenchymal cells into chondrocytes [4].

In conjunction with and as a possible consequence of this differentiation alteration, Ihh and Runx2, which stimulate the maturation of chondrocytes towards hypertrophy, showed a decrease in their protein levels (Figure 2). At this stage of the chondrogenic differentiation pathway, Bapx1 as well as Wnt5a were shown to stimulate *Ihh*, characterizing the prehypertrophic stage of the chondrocytes [18,28]. Similarly, Bmp7 is known to induce hypertrophy through *Runx2* stimulation [32]. The reduced protein levels of Bapx1, Wnt5a and Bmp7 may have resulted in a lower stimulation of *Ihh* and *Runx2*, leading to the observed lower protein accumulation and thereby explaining the decreased efficiency of the late maturation phase in endochondral ossification.

This Hoxa2 interference with both chondrogenic differentiation and the endochondral ossification cascade appears to be associated with a harmonious and proportionate short stature phenotype. On the other hand, the perturbation of either the proliferation or the hypertrophic stage by precise targeting of one or a combination of selected genes induces the formation of shorter but malformed skeletal elements. *Wnt5a* overexpressing mice, for example, feature a delay of chondrocyte hypertrophy and subsequent ossification, leading to a smaller but thicker appendicular skeleton [18]. In contrast, a regular hypertrophic development associated with a perturbed proliferation stage in *PthrP* knockouts induced a huge decrease in the proliferative column length and a slower cellular differentiation, leading to disproportionately short limbs with a correctly sized axial skeleton [41,42]. *Bmp7* knockout mice featured a reduction in the length of both limbs and trunk. However, their phenotype was supplemented by other malformations like extra-digits or a fusion of the neural arch [27]. *Runx2* deficient mice presented dwarfism but exhibited a deficit in calcification throughout the whole body, which is not the case for *Col2a1/Hoxa2-lacZ* [43]. When considering only the size reduction trait, *Wnt5a* knockouts exhibited a shorter but thicker skeleton [18,44]. Deficiencies in other actors were linked to a disproportionate short stature phenotype with a shortening of the trunk only in *Bapx1* knockouts [26,45] or solely the limbs in *Bmpr1a* [19,46], *Ihh* [47] or *Sox9* [48] loss-of-function. *Pax9* [49] and *Sox6* [50] deficient mice did not feature length reduction, despite the presence of an impressive phenotype when associated with a loss-of-function of their usual partners *Pax1* [51] or *Sox5* [52]. These examples of inappropriate skeletal development were directly related to the deregulation of one factor and their skeletal phenotype was never related to PSS syndrome.

In contrast, the present phenotype resulting from *Hoxa2* forced expression associates early differentiation disruption and proportionate short stature. Indeed, in the present mouse model, *Hoxa2* is expressed as soon as mesenchymal cells differentiate into chondrocytes, *i.e.*, concomitantly with Col2a1 expression, the marker of chondrogenesis initiation [3]. This failure is associated with a deregulation of protein levels whose combination induces a harmonious reduction of both the limbs and the vertebral column, a phenotype that is not observed upon individual chondrogenic gene misexpression. Although chondrogenesis is similar in the overall endochondral skeleton (*i.e.*, every skeletal element except some parts of the skull and face, the mandible, the scapula and the lateral part of the clavicle [53–57]), an analysis of the chondrogenic actors in *Col2a1/Hoxa2-lacZ* limbs should be performed to confirm that the differentiation cascade is impaired in a similar way in all endochondral skeletal pieces, either during the embryonic period and during postnatal growth.

Other chondrogenic factors behaved differently in the *Col2a1/Hoxa2-lacZ* gain-of-function. *Gdf* and *Msx* genes are also involved in the differentiation of mesenchymal cells into chondrocytes. Gdf5 and Gdf10 which positively control the differentiation of cells into chondrocytes *in vivo* and *in vitro* [58–60], are increased in *Hoxa2* overexpressing mice (Figure 2). On the other hand, Msx1, which has a negative influence on early chondrogenesis [31], is decreased. Other factors that are involved in the endochondral ossification process, such as Bmp4 [33], Gdf6 [34] and PthrP [16] showed no protein level differences between control and mutant mice. These results are not necessarily contradictory with the negative influence of *Hoxa2* over endochondral ossification given that a total loss of molecules involved in the chondrogenetic program would lead to a global deformity or absence of skeletal areas. Indeed, inactivation of *Sox9* using *Prx1-Cre* transgene induced a complete loss of cartilage and bone in mice limbs [61]. Moreover, *Runx2* null mice featured a total absence of calcified tissue leading to a boneless, cartilaginous skeleton except in the zeugopods [62].

Our data highlight a combined deregulation of distinct molecules under *Hoxa2* overexpression, which is schematized in Figure 2. This selective deregulation is responsible for an impaired endochondral skeleton, as observed in E18.5 transgenics [3], and is associated with a postnatal proportionate short stature phenotype [4]. While the misregulation of the chondrogenic factors highlighted here globally and concurrently contribute to the phenotype, the triggering event(s) that are under the immediate influence of Hoxa2 need to be identified. In particular, further experiments must be performed to highlight Hoxa2 direct targets and to decrypt the unknown interactions between the chondrogenic factors. Therefore, although no clinical correlation has been reported so far between the deregulation of a *Hox* gene and the appearance of human PSS, a similar impingement during the transition of mesenchymal cells to chondrocyte and the resulting misregulation in the phases of endochondral ossification may be considered to be involved in proportionate short stature pathogenesis. Moreover, other *Hox* genes could be involved in such misregulation since it was demonstrated that severe cartilage defects in *Hoxd4* and *Hoxc8* overexpressing mice were related to chondrogenic molecules impairment [63,64]. Indeed, during their RT-PCR experiments, Kruger and Kappen showed that *Mmps*, *Fgfs* and even *Ihh* or *Wnt5a* were altered in their transgenics [64].

## 3. Experimental Section

### 3.1. Transgenic Mice and Embryos

*Col2a1-Cre* [24] and *hβ-actin-lox-STOP-lox-Hoxa2-lacZ* [3] transgenic mice were mated in order to obtain animals with both transgenes so that *Hoxa2* expression was induced in *Col2a1* expressing territories and maintained thereafter, *i.e.*, throughout all of the endochondral elements of the skeleton. Animals carrying both transgenes are referred to as *Col2a1/Hoxa2-lacZ* or mutant and mice bearing only the *hβ-actin-lox-STOP-lox-Hoxa2-lacZ* transgene are referred to as *βS-Hoxa2-lacZ* or controls, for simplification purposes. Genotyping was performed by PCR, as described previously [4]. Experimental procedures were approved by the Animal Experimentation Ethics Committee of the Université catholique de Louvain (Ethical committee number 053001). Animal housing and handling were conducted according to the rules and regulations of the Belgian Federal State.

### 3.2. Immunohistochemistry

E13.5, E15.5 and E16.5 embryos (*n* = 5) were fixed in 4% formaldehyde for at least 24 h, embedded in paraffin and cut into 5 μm-thick sagittal slices. Sections were deparaffinized, rehydrated and were first treated for antigen retrieval with microwave exposure (750 W for 3 min and 4 cycles of 350 W for 3 min, 30 s) in a 0.01 M citrate buffer solution [65] containing 0.17% tritonX-100. Some sections were subsequently treated with 20 μg/mL Proteinase K (Sigma-Aldrich, St. Louis, MO, USA) in distilled water at 37 ºC for 5 min and then rinsed into 4 ºC distilled water for 5 min. Endogenous peroxidase was inhibited in all the tested sections using 1% H_2_O_2_ during 20 min and unspecific staining was blocked with 3% normal goat serum for 30 min. Samples were incubated overnight at room temperature with primary antibodies against Bapx1 (1:100), Runx2 (1:100), Sox5 (1:200), Sox6 (1:100), Bmpr1a (1:20), Foxc2 (1:10) (Abcam, Cambridge, UK), β1-integrin (1:40), Bmp7 (1:25), Gdf10 (1:10), Gdf5 (1:100), Ihh (1:50), Wnt5a (1:30) (R & D Systems, MS, USA), Bmp4 (1:50), Fgfr3 (1:100), Gdf6 (1:20), Meox1 (1:10), Meox2 (1:10), Pax1 (1:20), PthrP (1:100) (Santa Cruz Biotechnology Inc., Santa-Cruz, TX, USA), Msx1 (1:80) (Covance, NJ, USA), Msx2 (1:50) (United States Biological, Salem, MA, USA), Osteopontin (LF-124) (1:200) [66], Pax9 (1:100) (Genway, San Diego, CA, USA), S-100 (1:500) (ABR Affinity Bioreagents, Golden, CO, USA) and Sox9 (1:100) (Sigma-Aldrich, St. Louis, MO, USA). Thereafter, the sections were successively incubated with biotinylated Goat anti-rabbit, anti-mouse or Horse anti-goat secondary antibody (1:200) and treated with VECTASTAIN ABC Kit (Standard) (Vector laboratories, Burlingame, CA, USA) for 30 min each. Sections were stained with stable DAB (Life technologies, CA, USA) or EnVision^®^ + System–HRP (AEC) (Dako Denmark A/S, Glostrup, Denmark) for 3 or 5 min respectively, and finally counterstained in Mayer’s hematoxylin [67]. Unless mentioned, the sections were rinsed in 1% PBS/BSA solution after each described step. For comparison, the most stained cervical vertebra in each section, *i.e.*, the vertebra that featured the highest staining intensity and highest numberof stained chondrocytes, was photographed and considered for the experiment. Negative controls were obtained pre-incubating the antibodies with recombinant protein (according to manufacturer instructions). When unavailable, pictures were taken in an unstained area. Positive controls were verified on tissue specifically stained by the antibodies (Figure S1).

### 3.3. Western Blotting

Western blotting was performed as previously described [4]. Briefly, E13.5 (*n* = 5) total spines (from the first cervical to the last caudal vertebra) were dissected in PBS using sterile lancet and sonicated in 150 μL Laemmli buffer containing complete protease inhibitor cocktail (Roche, Indianapolis, IN, USA) for 3 × 30 s at frequency level 4, spaced with a 30 s resting period on ice using Vibracell 75022 Ultrasonic processor (Bioblock scientific, Illkirch, France). The whole protein extract was estimated using BCA Protein Assay Reagent (Pierce Biotechnology Inc., Rockford, IL, USA). After incubation at 95 ºC for 5 min, 30 μg samples were loaded on gel electrophoresis and transferred to nitrocellulose membrane. After overnight blocking in PBS Tween 0.1% containing 5% powdered milk, membranes were incubated overnight with Bapx1 (1:5000), Runx2 (1:2500), Sox5 (1:10,000), Sox6 (1:10,000), Bmpr1a (1:25,000) (Abcam, Cambridge, UK), Bmp7 (1:10,000), Gdf10 (1:1000), Gdf5 (1:500), Ihh (1:30,000), Wnt5a (1:10,000) (R & D Systems, Minneapolis, MN, USA), Bmp4 (1:10,000), Fgfr3 (1:5000), Gdf6 (1:100), PthrP (1:10,000) (Santa Cruz Biotechnology Inc., Santa-Cruz, TX, USA), Msx1 (1:10,000) (Covance, NJ, USA), Msx2 (1:10,000) (United States Biological, MA, USA), Pax9 (1:8000) (Genway, San Diego, CA, USA), β-actin (1:2000) and Sox9 (1:10,000) (Sigma-Aldrich, St. Louis, MO, USA) antibodies. After three washes in PBS Tween, membranes were incubated with biotinylated Goat anti-rabbit secondary antibody (1:200) and with VECTASTAIN ABC Kit (Standard) treatment (Vector laboratories, Burlingame, CA, USA) for 30 min each and were revealed using Pierce Supersignal West Pico trial kit (Pierce Biotechnology Inc., Rockford, IL, USA). The immunoblot band was processed for mean gray value measurement using Image J software (http://rsbweb.nih.gov/ij/index.html). Data normalization to measure the relative intensity of a given band was performed firstly after removal of the mean gray value of the background and then by reporting the measure obtained to that obtained for β-actin with the same sample. The data from *βS-Hoxa2-lacZ* and *Col2a1/Hoxa2-lacZ* groups were compared with unpaired Student’s *t* test. All data are presented as mean value (M) ± standard error of the mean (SEM). *p* < 0.05 was assigned as significant difference.

## 4. Conclusions

In conclusion, we propose a model that gives an insight into idiopathic proportionate short stature, displaying a decrease in proteins involved in the early phase of chondrocyte differentiation, *i.e.*, the mesenchymal-to-chondrocyte step (Figure 2). This model involves a decrease in Bapx1, Bmp7, Bmpr1a, Ihh, Msx1, Pax9, Sox6, Sox9 and Wnt5a. Moreover, it is accompanied by a lowering of factors that are involved in the late differentiation phase of the endochondral ossification, *i.e.*, Ihh and Runx2. The selective deregulation of these particular factors distributed along the chondrogenic pathway is likely to result in proportionate and harmonious, yet smaller skeletal growth.

## Supplementary Information
