# Supplementary material for: Molecular Study of a Hoxa2 Gain-of-Function in Chondrogenesis: A Model of Idiopathic Proportionate Short Stature
Source: Int J Mol Sci. 2013 Oct 14;14(10):20386–98. doi: 10.3390/ijms141020386 (PMC3821620; doi:10.3390/ijms141020386)
Supplement: Supplementary file 1 [file ijms-14-20386-s001.pdf]

Supplementary Information

Figure S1. Negative and positive controls used in immunohistochemical staining. For each molecule analyzed, the negative control (upper left) was a vertebral body after specific antigen pre-incubation. An unstained area was used when the peptide was unavailable. Positive controls were considered in stained territories (according to the literature).

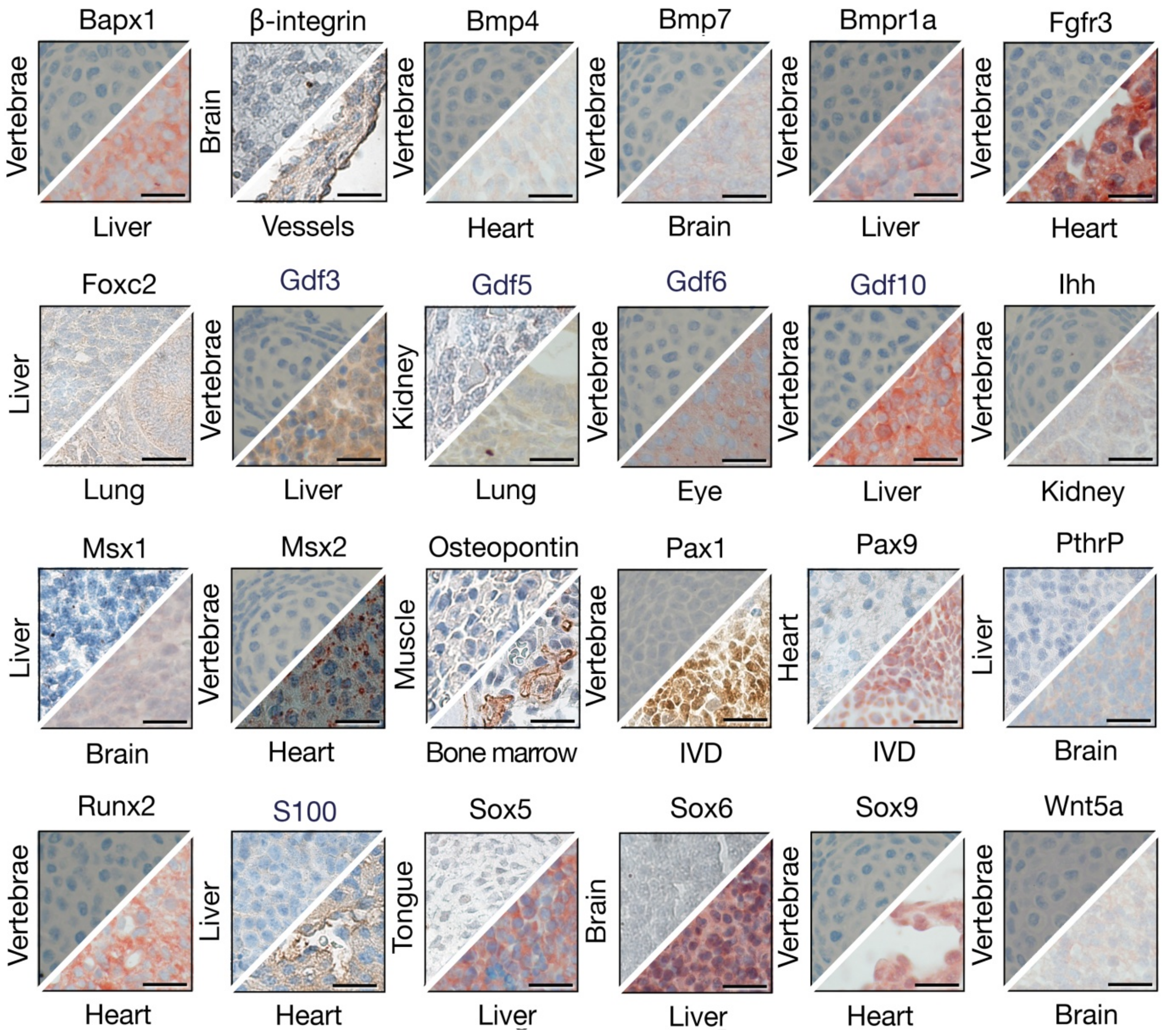

© 2013 by the authors; licensee MDPI, Basel, Switzerland. This article is an open access article distributed under the terms and conditions of the Creative Commons Attribution license (http://creativecommons.org/licenses/by/3.0/).
